# Entomopathogenic Nematode Compatibility with Vineyard Fungicides

**DOI:** 10.2478/jofnem-2023-0057

**Published:** 2023-11-23

**Authors:** Raquel Campos-Herrera, Elizabeth Carpentero, Miguel Puelles, José Luis Ramos Sáez de Ojer, Rubén Blanco Pérez

**Affiliations:** Departamento de Viticultura, Instituto de Ciencias de la Vid y del Vino (CSIC, Gobierno de La Rioja, Universidad de La Rioja), Finca La Grajera, Logroño, Spain; Servicio de Investigación Agraria y Sanidad Vegetal, Gobierno de La Rioja, Finca La Grajera, Logroño, Spain

**Keywords:** agrochemicals, grapevine, *Steinernema*, virulence, biological control

## Abstract

Vineyards, covering over seven million hectares worldwide, hold significant socio-cultural importance. Traditionally reliant on conventional practices and agrochemicals, this agroecosystem faces environmental challenges, including soil and water pollution. Sustainable viticulture, driven by eco-friendly practices and cost reduction, has gained prominence, underlining the importance of biological control agents such as entomopathogenic nematodes (EPNs). EPNs naturally occurr in vineyard soils and play a crucial role in controlling pest damage. Ensuring compatibility between EPNs and the commonly used vineyard fungicides is critical, as these applications constitute the predominant pest-management practice during the productive grapevine cycle.

This study assessed the impact of authorized grapevine fungicides on EPNs, focusing on the survival of populations and sublethal effects on their virulence. We investigated the compatibility of two EPN populations (*Steinernema feltiae* 107 and *S. carpocapsae* ‘All’) with three organic production-approved products (*Bacillus pumilus*, sulfur, and copper oxychloride) and two synthetic chemicals (Trifloxystrobin and Mancozeb). Our findings revealed that the viability of *S. feltiae* 107 was reduced when exposed to sulfur and copper oxychloride, and its virulence was affected by copper oxychloride and Mancozeb, although only two days after exposure and with no significant differences for larval mortality at five days.

In contrast, *S. carpocapsae* ‘All’ exhibited full compatibility with all five fungicides, with no impact on its viability or virulence. Consequently, our results suggested that the evaluated fungicides could be co-applied on both EPN populations if they were employed on the same day. However, further research on multi-target interactions is needed to ensure the successful implementation of this kind of co-application.

Grapevine, *Vitis vinifera* (Vitales: Vitaceae), a globally cultivated plant, holds great importance, covering a vast expanse of 7.25 million hectares and yielding 79.4 million tons of grapes worldwide in 2022 (OIV, 2023). This robust presence underscores viticulture's profound socioeconomic and cultural significance, especially in nations with solid wine-producing traditions, such as France, Spain, and Italy (OIV, 2023). Furthermore, the cultivation of grapevines has transcended its traditional Mediterranean climate domains, extending into temperate regions, including semidesert areas (Daane et al., 2018). This expansion has further elevated its significance in countries like China and the USA (OIV, 2023).

In traditional agriculture, vineyards have long stood as one of the most intensively managed crops, often characterized by the widespread application of synthetic agrochemicals (Nicholls et al., 2008; Winter et al., 2018). Unfortunately, practices of this kind have contributed significantly to pressing environmental issues, including alarming soil and water pollution (Pose-Juan et al., 2015; Herrero-Hernández et al., 2017). In response to these environmental and health concerns, more emphasis is being placed on integrated production methodologies, notably the adoption of Integrated Pest and Disease Management (IPDM). These methods have found their place within viticultural practices – for instance, under European Union regulation (EC, 2009). IPDM's objective is to maintain pest, disease, and weed growth below economically damaging thresholds through a judicious combination of legal, biological, cultural, biotechnological, and chemical measures (Barzman et al., 2015; Pecenka et al., 2021).

In pursuing viticulture sustainability, the focus is on implementing environmentally-friendly management tools, accompanied by a concerted effort to reduce the overall inputs borne by grape growers, thus curbing costs (Provost and Pedneault, 2016). Moreover, with the surging interest in organic viticulture, strongly promoted by numerous stakeholders and policymakers such as the EU as part of the European Grenn Deal (European Commission, 2020), the use of biological control agents is poised to assume an active role in the arsenal of management tools.

Regulation (EU) 2015/408 has set forth a chemical substitution list targeting the chemicals employed in industrialized agriculture. This initiative has catalyzed the advancement of novel physical, chemical, and biological control approaches as alternative pesticide solutions (EU, 2015). Regardless, contemporary on-farm pest and disease management strategies frequently blend non-chemical and chemical control methodologies (Damalas, 2009), so the pivotal challenge continues to be the compatibility of biological control agents with other products, especially agrochemicals.

Entomopathogenic nematodes (EPNs) are well-known biocontrol agents naturally occurring in crop soils, including vineyards (Lacey et al., 2015; Lewis et al., 2015; Blanco-Pérez et al., 2022). Their remarkable capability to kill a broad range of arthropods within a short timeframe (about 48–72 hours post-infection) qualifies them as an excellent non-chemical alternative for combating numerous insect pests (Kaya et al., 2006; Dillman et al., 2012; Dolinski et al., 2012; Lacey et al., 2015). Their highly resilient, infective juvenile (IJ) stage searches for a suitable host. Once located, IJs penetrate the host and release symbiotically associated bacteria in the hemocoel (Stock, 2015). Both nematodes and bacteria counter host defenses and reproduce until resources are depleted (Boemare, 2002; Bode, 2009). A new generation of IJs subsequently emerges from host cadavers to renew the life cycle.

Although research on using EPNs as biocontrol agents in vineyard agroecosystems remains an area yet to be exhaustively explored, numerous studies have begun to delve into their applications against a range of aerial pests plaguing vineyards (Campos-Herrera et al., 2021). Potential targets include *Planococcus ficus* (Hemiptera: Pseudococcidae), *Philaenus spumarius* (Hemiptera: Aphrophoridae), *Thaumatotibia leucotreta*, *Lobesia botrana* (Lepidoptera: Tortricidae), and *Vitacea polistimorfis* (Lepidoptera: Sessidae) (Williams et al., 2010; Vieux and Malan, 2015; Steyn et al., 2021; Vicente-Díez et al., 2021a, 2021b; Campos-Herrera et al., 2023). However, for this promising approach to materialize, it is imperative to consider the compatibility of specific EPN populations with the common agrochemical applications concurrently employed in vineyard management. Numerous studies have pursued this research goal over the past decades (Krishnayya and Grewal, 2002; Gutiérrez et al., 2008; Nermut and Mracek, 2010; Laznik et al., 2012; Laznik and Trdan, 2014; Can Ulu et al., 2016; Nalinci et al., 2021), with some of these investigations have suggesting that EPN-pesticide compatibility depends on nematode populations, agrochemicals, and application doses (De Nardo and Grewal, 2003; García del Pino and Jové, 2005; Laznik et al., 2012; Özdemir et al., 2020; Kotsinis et al., 2023).

It is also crucial to acknowledge that indications of compatibility go beyond the mere survival of IJs, as sublethal effects, including diminished virulence and reproductive capabilities, may conceal the comprehensive impact of chemical exposure on the efficacy of EPN applications (García del Pino and Jové, 2005; Gutiérrez et al., 2008; Özdemir et al., 2020).

Fungicides are among the more commonly used phytosanitary products in combatting diseases that threaten vineyards (Pertot et al., 2017). On average, vineyards often require approximately 12–15 fungicide treatments annually, even exceeding 25 treatments in dire years, to control harmful diseases like the oomycete *Plasmopara vitícola* (Peronosporales: Peronosporaceae), the fungi *Erysiphe necator* (Erysiphales: Erysiphaceae), and *Botrytis cinerea* (Helotiales: Sclerotiniaceae) (Pertot et al., 2017). If co-applications prove feasible, such a high treatment frequency could be partially exploited to apply EPN-based treatments, optimizing management strategies to improve overall efficacy and reduce costs.

In this study, we hypothesized that certain fungicides authorized for use in grapevine may exert lethal and/or sublethal effects on the effectiveness of EPNs as biocontrol agents, an effect that could also be contingent on the species/populations evaluated. The primary objective of this investigation was to assess the compatibility of a selection of commonly used vineyard fungicides with two specific populations of EPN species. Within this study, we investigated both the survival of EPNs exposed to fungicides for periods of 4 and 24 hours, and the sublethal effect on EPN virulence, as observed over durations of two and five days. We additionally explored the capability of fungicides to kill larvae of the model insect *Galleria mellonella* (Lepidoptera: Pyralidae) to ensure that any larval mortality resulted from EPN virulence rather than any unexpected direct fungicidal effect.

## Material and Methods

### Nematodes, insects, and fungicides

This study evaluated two EPN populations, specifically *Steinernema feltiae* 107 (GenBank Accesion number MW480131) and *S. carpocapsae* All (Genbank Accesion number MW574913). These two species naturally occur in Riojan vineyards in Northern Spain (Blanco-Pérez et al., 2020, 2022). Nematodes were routinely cultured using the last instar of *G. mellonella* as hosts. Nematode cultures were incubated at room temperature (∼22°C, 60% relative humidity, RH, without photoperiod). IJs were harvested from host cadavers in tap water and stored at 14 °C in darkness until use. IJs were employed in each experiment and trial at approximately two weeks upon emergence. The insect *G. mellonella* was reared following the procedure and feeding regimen described by Vicente-Díez et al. (2021a) and conducted in a growth chamber at 28 °C, 10% RH, and without photoperiod. The last instars of *G. mellonella* were used for the nematode rearing and subsequent experimental procedures.

We evaluated five fungicides authorized for use in Spanish vineyards (Table 1). Among those, *Bacillus pumilus* (Bacillales: Bacillaceae), sulfur, and copper oxychloride have received approval for utilization in organic production, whereas Trifloxystrobin and Mancozeb are exclusively recommended for IPDM practices. All products were promptly prepared upon their arrival at the laboratory to ensure optimal experimental conditions. Following procedures described by Campos-Herrera et al. (2023), each agrochemical was prepared by doubling the highest recommended field dose for a final volume of 0.5 L. Subsequently, each preparation was combined with the nematode-adjusted concentration in a 1:1 ratio, yielding a final fungicide concentration equal to the maximum field application dosage. All the experiments were repeated with a new mixture of the product and nematode preparations.

**Table 1:** Compatibility test of co-application of fungicides certified for use in vineyards in Spain with entomopathogenic nematodes.

**Commercial product**	**Supplier**	**Active ingredient**	**Concentration prepared**	**Recommended field application**
Sonata	Bayer	*Bacillus pumilus* strain QST 2808	20 ml/l	10 ml/l
Sulfur	Sipcam Jardin	Sulfur 80%	10 g/l	5 g/l
Copper oxychloride	Sipcam Jardin	Copper oxychloride 50% p/p	8 g/l	4 g/l
Flint	Bayer	(WG) with 50% trifloxystrobin	0.30 g/l	0.15 g/l
Ridomil-Gold	Syngenta	64%p/p of Mancozeb,3.9% of Metalaxyl-M	5.0 g/l	2.5 g/l

### Viability test:

The experimental procedure followed the methodologies described by Campos-Herrera et al. (2023). A nematode concentration of 20 IJs/100 μL per EPN population was prepared to investigate nematode survival following agrochemical exposure. For each fungicide, we mixed 8 ml of nematode suspension with 8 ml of the corresponding product, previously prepared at double the recommended field concentration (Table 1), and thoroughly mixed for one minute to ensure complete homogenization. For each EPN species test, the treatments (n = 3) were as follows: Control (water), *Bacillus pumilus,* (Bayer, Barcelona, Spain) sulfur, copper oxychloride (Sipcam Jardín, Valencía, Spain), Trifloxystrobin, (Bayer, Barcelona, Spain) and Mancozeb (Syngenta, Basel, Switzerland). We used 30-mm-diam. Petri dishes containing 1.5 ml of the corresponding nematode-product/control mixture. Petri dishes were maintained under controlled conditions at 22 °C, 60% RH, without photoperiod. Evaluations were conducted at 4 and 24 h post-exposure by counting all individuals with a stereomicroscope. Nematodes that displayed no movement after being gently touched three times with a specialized nematological needle were considered dead. The whole experiment was repeated with fresh material and preparations.

### Virulence test

To assess the sublethal effects of fungicidal exposure on EPN virulence, we evaluated its impact after 24 h of co-incubation, maintaining the conditions outlined in the viability test. As Campos-Herrera et al. (2023) proposed, we assembled 55-mm-diam. Petri dishes with Whatman no. 1 filter paper (Whatman, Maidstone, UK) covering both the bottom and top lids of the dishes. Then we added 250 μl of tap water on each side (n = 5 per treatment) and, without delay, the same volume of the nematode-product/control preparations described above. Finally, five *G. mellonella* larvae were placed onto each plate to complete the setup.

All the dishes were incubated in a humid chamber. A tray filled with tap water was positioned within the chamber, along with a cover encompassing all the dishes. This arrangement ensured the maintenance of adequate moisture throughout the experiment. The whole experiment was then repeated with new/fresh material and preparations.

### Mortality test

In addition to assessments involving nematodes, we evaluated the potential impact of the fungicides on insect larvae. This evaluation followed the previous identical procedure, except no nematodes were introduced. Instead, a control treatment consisting exclusively of water was employed as the negative control. This supplementary analysis aimed to ascertain that none of the pesticides possessed unexpected insecticidal effects that could alter the outcomes of the virulence test. The whole experiment was then repeated with new/fresh material and preparations.

### Statistical analysis

We conducted mixed linear model (MIXED) tests to assess the EPN viability and generalized mixed model (GLMM) tests, with a binomial distribution (logit-link function) for the virulence and mortality tests. In each model, we accounted for trails and blocks as random effects. All the statistical analyses were run using SPSS 27.0 (SPSS Statistics, SPSS Inc., Chicago, IL, USA). Descriptive statistics were presented as Least-Squares Means ± standard error of the mean.

## Results

### Viability test

**Figure 1:**
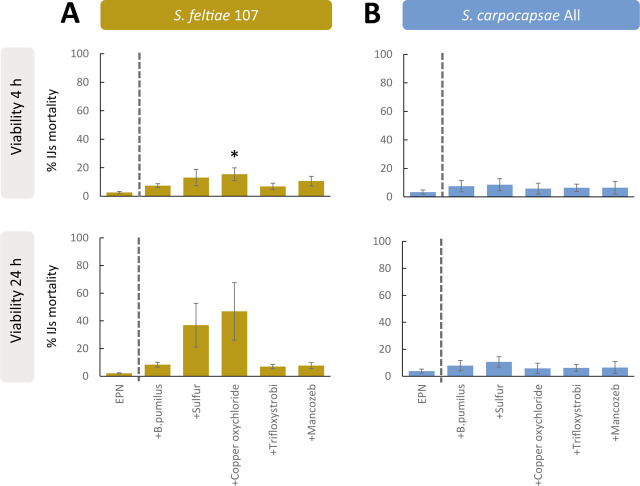
Viability of two entomopathogenic nematode (EPN) populations: (A) *Steinernema feltiae* 107 and (B) *Steinernema carpocapsae* All. Asterisks indicate significant differences at * *P* < 0.05 from mixed linear model testing within pair-treatment comparisons of single EPN applications and combined with five agrochemicals authorized for use in vineyards (see Table 1), after 4 and 24 h exposure. Values are least-square means ± SE.

Compared to the control treatment, copper and sulfur exposures exhibited apparent signs of increased EPN mortality among the evaluated fungicides, but only for the species *S. feltiae* (Fig. 1). Statistically significant differences were observed exclusively for copper oxychloride after 4 hours of exposure (F_1,10_ = 7.89; *P* = 0.018), though marginally significant differences were also reported for copper oxychloride (F_1,10_ = 4.87; *P* = 0.052) and sulfur (F_1,10_ = 4.67; *P* = 0.056) after 24 hours of exposure (Fig. 1A).

### Virulence and mortality tests

**Figure 2:**
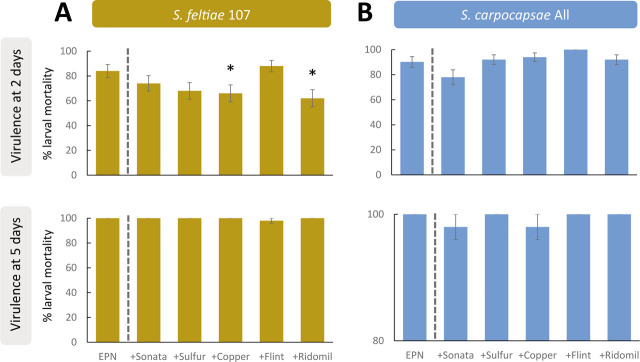
Virulence on *Galleria mellonella* larvae of two entomopathogenic nematode (EPN) populations: (A) *Steinernema feltiae* 107 and (B) *Steinernema carpocapsae* All. Asterisks indicate significant differences at *P < 0.05 from generalized mixed models testing within pair-treatment comparisons of single EPN applications combined with five agrochemicals authorized for use in vineyards (see Table 1), after two and five days of exposure. Values are least-square means ± SE.

**Figure 3:**
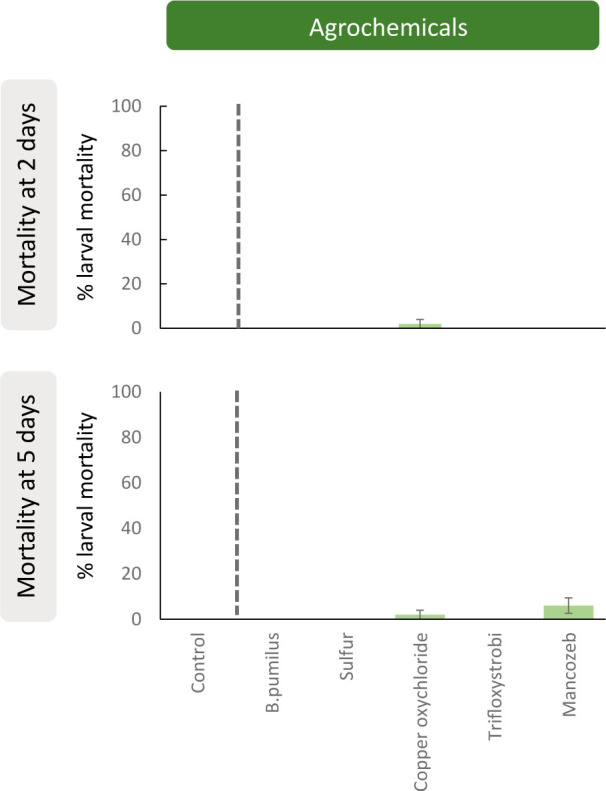
Mortality of *Galleria mellonella* larvae after two and five days after exposure to five agrochemicals authorized for use in vineyards (see Table 1). No significant differences were found from generalized linear mixed models testing within pair-treatment comparisons of control treatments and the agrochemical applications. Values are least-square means ± SE.

Sublethal fungicide effects were only found for the EPN species *S. feltiae* (Fig. 2). Specifically, compared to single IJ applications, copper oxychloride (χ^2^ = 0.59; *P* = 0.045) and Mancozeb (χ^2^ = 0.71; *P* = 0.014) significantly reduced *S. feltiae* virulence after two days of exposure, but there was no significant difference after five days of exposure (Fig. 2A). No larval mortality was observed for the control treatments (*P* < 0.001; data not shown). Regarding the mortality test, none of the fungicides caused a significant increase in larval mortality when individually applied to insects (Fig. 3).

## Discussion

We observed that only specific fungicides authorized for grapevine use had discernible adverse effects, and only for one nematode species, *S. feltiae.* Notably, this EPN species has previously been described as particularly sensitive when interacting with agrochemicals of various types, including acaricides, insecticides, herbicides, and fungicides (Nermut and Mracek, 2010). Conversely, De Nardo and Grewal (2003) reported a high level of compatibility between *S. feltiae* and a range of pesticides, including nine fungicides, even when assessed 72 h after mixing with the highest recommended doses. This contrast evinces plausible intraspecific variability, a possibility also documented for the other nematode species we tested, *S. carpocapsae.* In our study, the population *S. carpocapsae* ‘All’ demonstrated resilience, showing no negative impact on its viability or virulence when exposed to the tested products.

However, Can Ulu et al. (2016) offered a different perspective. Following a similar methodology to ours, they found that diverse fungicides significantly affected the *S. carpocapsae* DD-136 population. Specifically, the active compound Captan induced mortality rates of approximately 23%, while Fosety-al (Placate) raised IJ mortality to 53% in 24 h. These findings underscore the intricacies in understanding agrochemical impacts, indicating that they may be contingent on the specific nematode species and even population-dependent.

We observed noteworthy findings in the viability of nematodes that had been exposed to fungicides. Specifically, sulfur and copper oxychloride, both approved for use in organic production, were associated with an increase in IJ mortality in *S. feltiae* (both marginally significant, *P* < 0.06, after 24 hours exposure). These outcomes align with previous research on the compatibility of fungicides with this particular EPN species. For instance, Özdemir et al. (2020), employing a similar methodology, reported that the chemical fungicides fluxapyroxad+difenoconazole and ametoctradin+dimethomorph resulted in an approximately 25% mortality rate among IJs in *S. feltiae.* Similarly, Nermut and Mracek (2010) documented high IJ mortality when native *S. feltiae* populations from Ustinov (Russia) were exposed to sulfur (Sulka, AgroBio, Brumovice, Czech Republic), with mortality exceeding 50%.

Nevertheless, despite their impact on IJ survival, it is worth noting that while copper oxychloride and Mancozeb exhibited a significant reduction in EPN virulence against *G. mellonella* larvae, they only did so for *S. feltiae* 107 after two days of exposure. This unexpected observation diverged from previous studies. For instance, Nermut and Mracek (2010) reported a 50% reduction in EPN virulence when exposed to fungicides. Similarly, Can Ulu et al. (2016) found significant reductions in the virulence of *S. carpocapsae* DD-136 when exposed to the fungicides Fosetyl-al and Captan, precisely 83% and 87%, respectively.

Regarding the co-exposure of EPNs with *B. pumilus*, we observed complete compatibility with both EPN populations, with no effect on nematode viability or virulence (Can Ulu et al., 2016). However, a challenging question still needs to be addressed: whether the presence of nematodes might influence the fungicidal effectiveness of the chemical product. Interactions between biological control agents can manifest differently, involving synergic, additive, or antagonistic effects, when targeting the same pest (Shapiro-Ilan et al., 2004; Acevedo et al., 2007; Wu et al., 2014). Our knowledge about the co-application of multiple biocontrol agents, each addressing diverse problems, still needs to be improved. Future research efforts must explore the intricate dynamics of the co-application of biocontrol agents and agrochemicals to ensure their compatibility and optimize their combined efficacy.

Our investigation has yielded encouraging results regarding the compatibility of the combined application of EPNs and fungicides. Specifically, neither the viability nor the virulence of the IJs belonging to the *S. carpocapsae* ‘All’ population were adversely affected by any of the tested agrochemicals. Conversely, we observed some adverse effects for *S. feltiae* 107 that nonetheless did not compromise its virulence five days post-exposure.

In practical terms, standard procedure involves preparing chemical or biocontrol products just before tractor-based application, all on the same day. Therefore, co-application becomes viable if nematodes remain viable and active in the presence of fungicides during the initial four hours of exposure. This observation is particularly significant considering that *S. feltiae* and *S. carpocapsae* have been proposed as promising candidates for controlling aerial pests such as *L. botrana* (Campos-Herrera et al., 2023). Our results thus open up exciting possibilities for co-applicating these EPN species with commonly used fungicides, three of which are allowed in organic productions.

### Declaration of competing and conflict of interest

The authors declare that they have no known competing financial interests or personal relationships that could have appeared to influence the work reported in this paper.

### Archive of data

The data presented in this study will be archived at https://digital.csic.es/ to ensure we comply with the FAIR mandate and that results are fully accessible to any researcher.
